# ZIKV Infection Induces an Inflammatory Response but Fails to Activate Types I, II, and III IFN Response in Human PBMC

**DOI:** 10.1155/2018/2450540

**Published:** 2018-06-03

**Authors:** Francesca Colavita, Veronica Bordoni, Claudia Caglioti, Mirella Biava, Concetta Castilletti, Licia Bordi, Serena Quartu, Marco Iannetta, Giuseppe Ippolito, Chiara Agrati, Maria Rosaria Capobianchi, Eleonora Lalle

**Affiliations:** ^1^Laboratory of Virology, National Institute for Infectious Diseases “L. Spallanzani” IRCCS, Via Portuense 292, 00149 Rome, Italy; ^2^Cellular Immunology Laboratory, National Institute for Infectious Diseases “L. Spallanzani” IRCCS, Via Portuense 292, 00149 Rome, Italy; ^3^Clinical Department, National Institute for Infectious Diseases “L. Spallanzani” IRCCS, Via Portuense 292, 00149 Rome, Italy; ^4^Department of Epidemiology, National Institute for Infectious Diseases “L. Spallanzani” IRCCS, Via Portuense 292, 00149 Rome, Italy

## Abstract

The recent epidemic in the Americas caused by Zika virus (ZIKV), Asian lineage, spurred the research towards a better understanding of how ZIKV infection affects the host immune response. The aim of this study was to evaluate the effects of Asian and East African ZIKV strain infection on the induction of IFN and proinflammatory and Th2 cytokines in human PBMC. We reported a slight modulation of type II IFN in PBMC exposed to Asian strain, but not to African strain, and a complete lack of type I and III IFN induction by both strains, suggesting the ability of ZIKV to evade the IFN system not only inhibiting the antiviral IFN response but also IFN production. Moreover, we highlighted a polyfunctional immune activation only in PBMC exposed to Asian strain, due to the induction of an inflammatory profile (IL-6, IL-8) and of a Th9 (IL-9) response. Overall, our data show a different ability of the ZIKV Asian strain, with respect to the African strain, to activate host immune response that may have pathogenetic implications for virus spread *in vivo*, including mother-to-child transmission and induction of severe fetal complications, as birth defects and neurological disorders.

## 1. Introduction

Zika virus (ZIKV) is an emerging mosquito-borne flavivirus, transmitted by different *Aedes* mosquito species, including *Aedes aegypti*. ZIKV tropism is various, and in addition to both skin fibroblasts and epidermal keratinocytes, dendritic cells (DCs) were found to be permissive to infection [1]. ZIKV was originally identified in Uganda in 1947 [2] and over the years limited ZIKV outbreaks were reported, especially in the Pacific islands [3, 4]. Phylogenetic studies have revealed that Zika virus has evolved into 3 distinct genotypes: West African (Nigerian cluster), East African (MR766 prototype cluster), and Asian [5]. In 2015, the largest ZIKV epidemic has begun in Brazil, spreading throughout the Americas and infecting nearly two million people; the virus that caused this epidemic was found to be most closely related to a 2013 isolate from French Polynesia, within the Asian clade [6]. Although ZIKV-associated illness was initially described as self-limiting mild illness characterized by rash, fever, conjunctivitis, arthralgia, and arthritis [7], it is now generally accepted that ZIKV is responsible for neurological birth defects, including microcephaly in newborns [8].

The innate immune response and, in particular the interferon (IFN) system, plays a key role in orchestrating protection against flaviviruses infection. However, the role of the innate immune response in ZIKV infection is controversial. A few studies have reported the ability of placental macrophages and primary human trophoblasts to limit ZIKV infection by producing type I and III (especially IFN-*λ*1) IFN, respectively [9, 10]. Furthermore, it has been shown that the exposure of primary human fibroblasts to ZIKV triggers IFN-*β* response, accompanied by the activation of type I IFN signaling pathways and the expression of several antiviral genes [1]. On the contrary, Kumar and colleagues observed a strong suppression of IFN and ISG expression in ZIKV-infected cells *in vitro* and a significant reduction in type I and type III IFN signaling [11]. In addition, the inhibition of type I IFN protein translation was observed during ZIKV infection in DCs, despite its induction at a transcriptional level. Furthermore, treatment of human DCs with retinoic acid-inducible gene I (RIG-I) agonist was shown to potently restrict ZIKV replication, while type I IFN had only modest effects [12]. A recent study on mammal cells demonstrated that ZIKV NS5 protein is able to activate IFN-*γ* in a selective manner while having an opposite effect on type I and III IFNs, namely, suppressing their activity. Therefore, this selective activation of IFN-*γ* signaling by ZIKV might also have an impact on other IFN-*γ*-regulated immune functions, such as macrophage activation and Th1 response [13]. Moreover, an aberrant activation of innate immunity has been observed during the acute phase of ZIKV-infected patients leading to a cytokine profiles associated with Th1 (IL-2 and nonsignificantly IFN-*γ*), Th2 (IL-4, IL-13), Th17 (IL-17), and also Th9 (IL-9) responses [14]. In particular, a modulator role of IL-9 on antiviral immunity has been suggested in human viral infections, that is, an association of elevated IL-9 levels and severe acute respiratory syncytial virus infections [15] and nonresponse to treatment in chronic hepatitis C [16] has been described.

To date, infection differences between African and contemporary Asian lineages have been scarcely investigated and the current outbreak spurred the research towards a better understanding of how ZIKV infection affect the host immune response. In this study, we evaluated the effects of Asian (INMI1, GenBank KU991811.1) and East African (MR766, GenBank LC002520.1) ZIKV strains infection on the induction of IFN, proinflammatory, and Th2 cytokines in human PBMC.

## 2. Materials and Methods

### 2.1. Virus Stock Preparation

Vero E6 cells (ATCC® number CRL-1586™) were maintained in Modified Eagle Medium (MEM) supplemented with 10% heat-inactivated fetal calf serum (FCS) at 37°C in a humidified atmosphere of 5% CO_2_. For virus stock preparation, Vero E6 cells were infected with ZIKV strains MR766 (provided by the European virus archive, EVAg) and the 2016/INMI1 (INMI1) isolate (GenBank Accession number KU991811) obtained from a traveler returning from Brazil in January 2016. Cell lysates were clarified, aliquoted, and stored at −80°C until use. Virus titration was performed on Vero E6 cell line by limiting dilution assay; the titer was calculated using the method of Reed and Muench and expressed as tissue culture infectious dose TCID_50_/mL [17]. Virus stock titers were 10^7.37^ TCID_50_/mL for MR766 and 10^6.12^ TCID_50_/mL for INMI1.

### 2.2. PBMC Infection

PBMCs were obtained from healthy donors by Ficoll/Hypaque (Pharmacia, Sweden) density centrifugation. Cultures were performed in RPMI 1640 medium (GIBCO, USA) containing 10% heat-inactivated FCS. PBMCs from 4 donors were used in the infection experiments. For these experiments, fresh PBMCs were exposed to either MR766 or INMI1 for 1 hour at 37°C with different multiplicity of infection (MOI), ranging from 0.1 to 10 TCID_50_/mL; at the end of the adsorption period, PBMCs were washed, reseeded at 2 × 10^6^ cells/mL in RPMI 10% FCS, and incubated at 37°C. At 24, 48, and 72 hours postinfection (hpi), supernatants and cells were collected and stored at −80°C for subsequent analysis.

### 2.3. Detection of ZIKV Replication

To assess viral replication, total RNA was extracted from cells by TRIzol (Life Technologies, NY, USA), according to the manufacturer's protocol, and ZIKV RNA was amplified using real-time RealStar Zika Virus RT-PCR Kit (Altona Diagnostics, Hamburg, Germany) in a Rotor-Gene Q Real-Time cycler.

### 2.4. IFN and Cytokines Detection

IFN-*α* and IFN-*λ* released in PBMC supernatants were measured by enzyme-linked immunosorbent assay (ELISA), namely, VeriKine-HS Human IFN-*α*, purchased from PBL Assay Science (Piscataway, NJ, USA) and DuoSet Human IFN-*λ* 1/3 (IL-29/IL-28B), purchased from R&D System Inc. (Minneapolis, MN, USA). Results were expressed as pg/mL. The detection range was 1.95–125 pg/mL for IFN-*α* and 62,50–4000 pg/mL for IFN-*λ*. As a positive control for IFN induction, Newcastle disease virus (NDV) was used in all the experiments, at 10 hemagglutination units (HU)/10^6^ cells. As a negative control for cytokine detection, PBMCs treated with only medium were used.

Supernatants were assayed by using the multiplex bead-based assays Bio-Plex Pro Human group I [IL-4 (low limit of quantification LLOQ: 0.16 pg/mL), IL-6 (LLOQ: 0.56 pg/mL), IL-8 (LLOQ: 0.35 pg/mL), IL-9 (LLOQ: 0.18 pg/mL), IL-10 (LLOQ: 1.3 pg/mL), IFN-*γ* (LLOQ: 3.08 pg/mL), and TNF-*α* (LLOQ: 1.9 pg/mL), Bio-Rad Laboratories, CA, USA]. Plates were measured using the Bio-Plex MagPix System and analyzed with the Bio-Plex Manager version 6.0 (Bio-Rad Laboratories, CA, USA).

### 2.5. Statistical Analysis

Results are expressed as median. Statistical significance was assessed by Mann–Whitney *U* test by using GraphPad Prism 5.0 (GraphPad Software Inc., La Jolla, CA, USA). A *p* < 0.05 was considered statistically significant.

### 2.6. Ethics Statement

The Institutional Ethics Board of INMI approved the use of PBMC collected from healthy donors for research purposes. Healthy donors signed a specific informed consent for any single procedure or treatment performed, after a thorough explanation of reasonably anticipated benefits and potential hazards of intervention and for the publication of the research study.

## 3. Results

### 3.1. Both MR766 and INMI ZIKV Strain Failed to Induce IFN in Human PBMC

To test the ability of ZIKV to induce IFN response in human PBMC, cells from healthy donors were exposed to different ZIKV strains (either East African MR766 or the contemporary Asian INMI1) at a MOI 0.1. No productive replication of both ZIKV strains was observed. NDV was used as a positive control of IFN induction: in all cases, a strong response was observed in NDV-exposed PBMC. Specifically, NDV induced IFN-*α* [24 h median 122.7 pg/mL (107.8–142.5); 48 h: 119.4 pg/mL (107.8137.4); 72 h: 121.4 pg/mL (109.1–133.8)], IFN-*γ* [24 h median: 54.7 pg/mL (42.8–58.5); 48 h: 42.5 pg/mL (32.7–56.5); 72 h: 67.1 pg/mL (37.3–86.6)], and IFN-*λ* [24 h median: 1650.0 pg/mL (411.6–2202.0); 48 h: 1539 pg/mL (442.5–2000.0); 72 h: 512.7 pg/mL (374.0–607.4)], indicating a good inducibility of IFN response in our experimental conditions.

**Figure 1:**
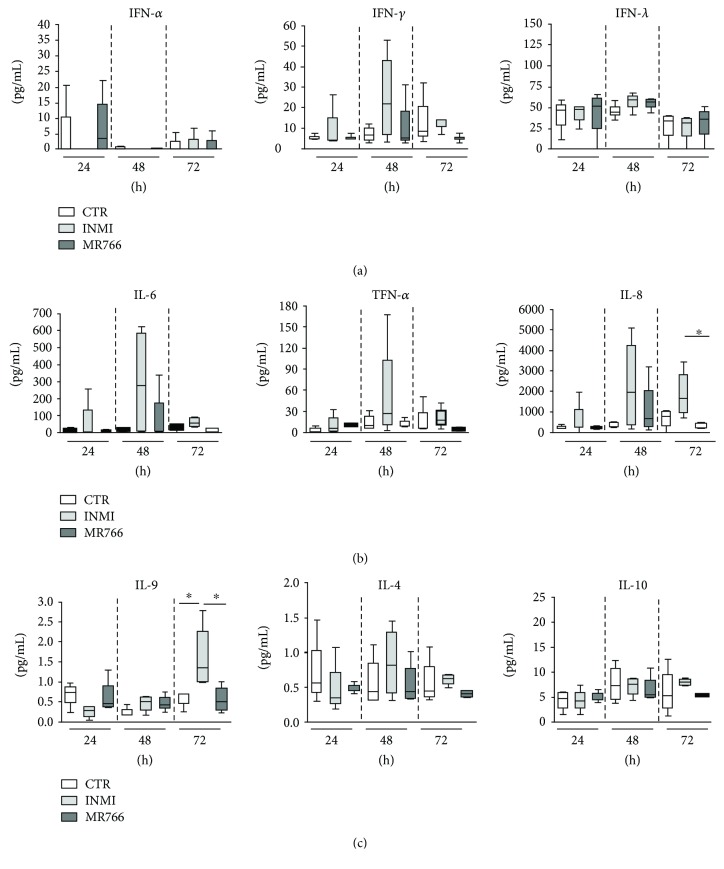
Activation of IFN (a) and cytokine (b and c) response in supernatants of PBMC after 24, 48, and 72 hpi at MOI 0.1 TCID_50_/mL either with MR766 or INMI1. The results of four independent experiments are expressed as pg/mL. Mann–Whitney *U* test, ^∗^*p* < 0.05.

Supernatants of infected PBMC were collected at different hpi (24, 48, and 72), and the production of different IFN types was assessed. No activation of either type I or III was observed after exposure to either ZIKV strains. Only type II IFN (IFN-*γ*) showed, although not significant, a slight increase in response to INMI1 infection with a peak at 48 hpi but not to MR766 (Figure 1(a)). It has to be considered that none of the fourth donors had a previous contact with any other flavivirus, since sera tested by indirect immune fluorescence assays (IFA) for Zika, dengue, and yellow fever were all below the detection threshold (1/20).

Moreover, even using higher MOI (1 and 10) to infect PBMC, neither evidence of viral replication nor induction of IFN system was observed (data not shown).

### 3.2. INMI ZIKV Strain Induces Proinflammatory Response in Human PBMC

To evaluate the ability of ZIKV to induce an inflammatory response in PBMC, cytokine production was quantified after challenge with INMI1 and MR766 ZIKV strains. In particular, inflammatory (IL-6, IL-8, and TNF-*α*), anti-inflammatory (IL4 and IL-10), and Th9 (IL-9) cytokines were evaluated in response to either ZIKV strain infection (Figures 1(b) and (c)). The analysis of IL-6, IL-8, TNF-*α*, and IL-4 showed a large variability mainly after 48 hpi. A transient but not significant induction of IL-6 and TNF-*α* was observed 48 hpi after INMI1 but not after MR766 exposure (Figure 1(b)). Differently, IL-8 was induced at 48 hpi after INMI1 exposure and persisted at 72 hpi. Notably, a significant difference between INMI1 and MR766 was detectable at 72 hpi, being IL-8 levels higher for INMI1 with respect to MR766 (*p* = 0.02) (Figure 1(b)). The levels of the Th9 response indicate a significant induction of IL-9 after INMI1 infection which peaked at 72 h after INMI1 infection (*p* = 0.02). In contrast, no significant upregulation of IL-9 protein was evidenced following MR766 infection (Figure 1(c)). Finally, no anti-inflammatory response was observed after INMI1 and MR766 infections (Figure 1(c)).

## 4. Discussion

Following the recent outbreak raging in the Americas, ZIKV has become an emerging arbovirus with potential impact on human health, as supported by the WHO Declaration of PHC in February 2016 [18]. To our knowledge, limited and controversial information is available about the innate host immune response developed in response to ZIKV infection in humans. All vector-borne flaviviruses studied so far need to overcome type I IFN signaling to replicate and cause disease in vertebrates [19] and the mechanisms by which ZIKV interferes with the innate response are the object of intense investigation [20] and are still to be fully understood.

In this paper, we analyze the ability of ZIKV to activate immune response in human PBMC, since it is well known that these cells represent the gatekeeper of virus spread in the body during systemic viral infections and include several subpopulations that may cooperate in the activation process [21, 22].

Our results show that ZIKV failed to induce both type I and type III IFN while is able to poorly activate type II IFN and to produce most proinflammatory cytokines such as IL-6, IL-8, and IL-9. In particular, we reported a slight modulation of type II IFN in PBMC exposed to INMI1 strain, but not to MR766, and a complete lack of type I/III IFN induction by both strains suggesting the ability of ZIKV to evade the IFN system not only inhibiting the antiviral IFN response [13] but also the IFN production. Moreover, we highlighted a polyfunctional immune activation only in PBMC exposed to INMI1 strain, due to the induction of an inflammatory profile (IL-6, IL-8, and TNF-*α*) and of a Th9 (IL-9) response. IL-6 as well IL-8 is associated with the acute-phase response and represents key inflammatory signals to induce an effective immune response as they are responsible for the activation of cellular components of the innate response and coordinate the T-lymphocyte proliferation. Nevertheless, during dengue infection, high levels of IL-6 and IL-8 are associated with severe/hemorrhagic infections [23, 24], suggesting that an unbalanced inflammatory response may participate to the pathogenesis of the disease. Of note, it has been recently reported in dengue infection that a coordinate balance between proinflammatory and regulatory signals represent the key for a protective immune response [24]. On the other side, IL-9 is a cytokine produced primarily by CD4+ Th9 cells and is generally reported to mediate allergic and autoimmune diseases [25]. Recent studies suggest that IL-9 plays an important role during human viral infection such as in severe acute respiratory syncytial virus infections [15] and in viral myocarditis [26]. The role of IL-9 in the modulation of both Th1 and Th2 T cell immunity as well as in the protection and/or pathogenesis of viral diseases needs further investigations.

Our observation is in line with Tappe et al.'s study that reported an upregulation of inflammatory cytokines levels and activation of type II IFN in human serum samples during acute ZIKV infection; in particular, IFN-*γ* showed a poor increasing trend in the acute and recovery phase [14], extending the knowledge on modulation of immune parameters during ZIKV infection. Moreover, in our experimental conditions, no productive replication was observed for both ZIKV strains, not even using high MOI (1 and 10), thus suggesting that membrane mechanisms could be responsible for IFN and cytokine induction as previously shown for other viruses, including HIV, HSV, and SARS [22, 27, 28].

In conclusion, our results indicated that neither strain is able to activate an efficient IFN response, except for a poor activation of type II IFN by INMI1 strain. Moreover, the induction of inflammatory cytokines was observed. The lack of activation of type I and III IFN response in PBMC may reflect an escape mechanism from the innate immune defense, which may be exceeded by the presence of other acute-phase proinflammatory cytokines, such as IL-6, IL-8, and IL-9. This peculiar behavior may have pathogenetic implications for virus spread *in vivo*, including mother-to-child transmission and induction of severe fetal complications, as birth defects and neurological disorders.

Further investigation is needed to establish a link between these observations and the correct activation of the immune system in ZIKV infection and to identify different intracellular signals induced by different ZIKV strains, responsible for different cytokine response.
